# The association between ambient temperature and preterm birth in Shenzhen, China: a distributed lag non-linear time series analysis

**DOI:** 10.1186/s12940-016-0166-4

**Published:** 2016-08-08

**Authors:** Zhijiang Liang, Yan Lin, Yuanzhu Ma, Lei Zhang, Xue Zhang, Li Li, Shaoqiang Zhang, Yuli Cheng, Xiaomei Zhou, Hualiang Lin, Huazhang Miao, Qingguo Zhao

**Affiliations:** 1 Department of Public Health, Guangdong Women And Children Hospital, 521, 523 Xing Nan Street, Panyu District, 511442 Guangzhou, Guangdong China; 2 Department of Children Health Care, Shenzhen Women and Children Hospital, Shenzhen, Guangdong China; 3 Department of Information Network, Meteorological Bureau of Shenzhen Municipality, Shenzhen, Guangdong China; 4 Department of Public Health, Fu Tian Maternal and Children Health Hospital, Shenzhen, Guangdong China; 5 Department of Public Health, Luo Hu Maternal and Children Health Hospital, Shenzhen, Guangdong China; 6 Department of Public Health, Long Gang Maternal and Children Health Hospital, Shenzhen, Guangdong China; 7 Department of Public Health, Bao An Maternal and Children Health Hospital, Shenzhen, Guangdong China; 8 Fu Tian Hospital of TCM, Shenzhen, Guangdong China; 9 Guangdong Provincial Institute of Public Health, Guangdong Provincial Center for Disease Control and Prevention, Guangzhou, Guangdong China

**Keywords:** Preterm birth, Ambient temperature, Time-series study, Distributed lag non-linear model

## Abstract

**Background:**

A few studies have examined the association between ambient temperature and preterm birth (PTB), and the results have been inconsistent. This study explored the association between ambient temperature and PTB in Shenzhen, China.

**Methods:**

Data of daily singleton PTB, air pollution and meteorological variables from 2005 to 2011 were collected in Shenzhen. A distributed lag non-linear model (DLNM) was used to investigate the association of the low and high temperatures (1st, 5th, 95th, and 99th percentiles) with PTB.

**Results:**

The median temperature was 24.5 °C and the 1st, 5th, 95th, and 99th percentiles of daily mean temperatures were 9, 12.5, 29.9 and 30.7 °C, respectively. The prevalence of singleton PTB was 5.61 % in Shenzhen. The association between temperature and PTB was not linear. There was an immediate positive association of low temperature (1st and 5th percentiles) and a negative association of high temperature (95th and 99th percentiles) with PTB. The effect of low temperature 9 °C (1st) on PTB on the current day was stronger than that of 12.5 °C (5th), with a relative risk (RR) of 1.54 (95 % CI: 1.36–1.75) and 1.49 (95 % CI: 1.35–1.63), respectively. The cumulative RR (up to 30 days) of 9 and 12.5 °C was 1.72 (95 % CI: 1.28–2.33) and 1.96 (95 % CI: 1.60–2.39), respectively. The cumulative effects (up to 30 days) of high temperature (95th and 99th percentiles) on PTB were 0.69 (95 % CI: 0.60–0.80) and 0.62 (95 % CI: 0.52–0.74), respectively. The cumulative effect (up to 30 days) of low temperatures on vaginal delivery PTB was lower than that of the cesarean section PTB with an RR of 1.58 (95 % CI: 1.12–2.22) and 1.93 (95 % CI: 1.21–3.08), respectively.

**Conclusions:**

This study suggests that low temperature might be a risk factor, while high temperature might be a protective factor of PTB in Shenzhen.

**Electronic supplementary material:**

The online version of this article (doi:10.1186/s12940-016-0166-4) contains supplementary material, which is available to authorized users.

## Background

Preterm birth (PTB), defined as any live birth before 37 completed weeks of gestation, is an important public health problem [1]. It was estimated that there were 15 million PTB worldwide in 2010 [2]. Globally, PTB is the leading cause of newborn deaths and the second largest direct cause of death only after pneumonia among children under five years, and over one million babies die annually from PTB-related complications [3].

The etiology of PTB remains unclear. Villar et al. proposed a PTB phenotype classification that incorporates five components-maternal conditions, fetal conditions, placental conditions, signs of parturition initiation, and the pathway to delivery [4]. The present findings suggest that the causes of PTB are a complex mix of genetic, behavioral, socio-economic and environment factors. Race [5, 6], maternal chronic infections and hypertension [7], maternal smoking [8, 9], maternal age [10–13], and air pollution [14, 15] all were identified as potential risk factors.

With the emerging interest in climate change and its health impacts, some researchers focused their studies on the association between the extreme high temperature and PTB. Although having been studied in some countries, the findings remained inconclusive. For instance, studies in the US [16, 17], Israel [18], Greece [19], Spain [20, 21], Australia [22, 23], Italy [24], and Sweden [25] reported a significant association between high temperature and PTB, but another US study [26] and some studies in England [27] and Germany [28] did not find any significant association between high temperature and PTB. The discrepancies may be due to the significant heterogeneities in the study design, population characteristics, and the data analysis of these studies.

The mechanism by which high temperature triggered PTB is unclear. There are several possible mechanisms. First, insufficient fluids in the mother due to high temperature can decrease the blood flow available to the fetus and induce uterine contractions [29]. Second, pregnant women may experience difficulty with thermoregulation and become dehydrated during the heat exposure, which may possibly result in a decrease in uterine blood flow and trigger labor [17]. Third, maternal heat stress may also trigger a release of hormones such as cortisol, which may in turn induce labor [18]. To our knowledge, few studies about the potential association between temperature and PTB have been carried out in China.

In the current study, we used a distributed lag non-linear time series analysis to explore the association between the ambient temperature and PTB in Shenzhen, China. The aim of this study is to increase the awareness of policy makers and clinicians regarding the role of temperature exposure on PTB in Shenzhen.

## Methods

### Study setting

**Fig. 1:**
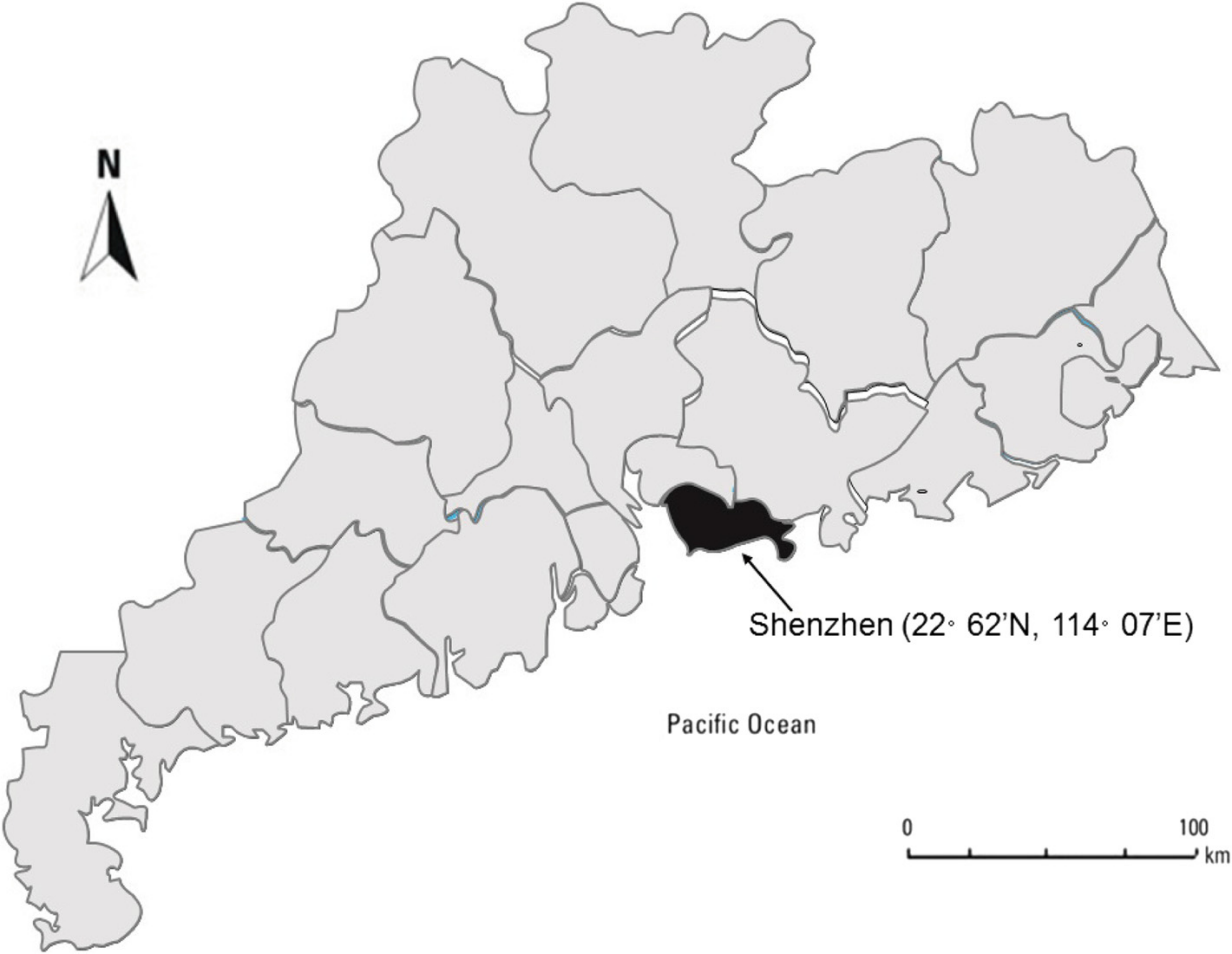
The map of Shenzhen

Shenzhen, located in southeastern China (22°27′ to 22°52′ north latitude), is a subtropical and coastal city; it has an area of 2000 km^2^, and belongs to subtropical oceanic monsoon climate with an average mean temperature of 23.0 °C. The mean daily temperature of the coldest month in January is 15.4 °C, and the mean daily temperature of the hottest month in July is 28.9 °C. The data of air pollution, meteorological factors and birth registry with high quality are available, thus, we chose Shenzhen as the study site. Figure 1 shows the location of Shenzhen in Guangdong Province, China.

### Data source

Daily data of preterm birth from January 1st, 2005 to December 31st, 2011 were collected from the birth registry database, which covered all midwifery clinics and hospitals in Shenzhen. Shenzhen maternal and children health information system has been constructed since 2000. One component of the system is birth certificate and all midwifery clinics and hospitals are using this system to report birth certificate through the network. The variables collected in birth registry database included date of birth, date of mother’s last menstrual period (LMP), birth weight, infant sex, maternal age, delivery mode, etc. Gestational age was computed as the number of weeks between the date of the last menstrual period (LMP) and the date of birth.

Daily meteorological data were obtained from the Shenzhen Meteorological Bureau websites. The variables included daily mean temperature (°C), relative humidity (RH, %), and atmospheric pressure (BP, hpa).

Daily 24-hour average air pollution data were collected from the Environmental Monitoring Center located in the center of Shenzhen. Air pollution data included particulate matter with an aerodynamic diameter less than or equal to 10 um (PM_10_, in ug/m^3^), sulfur dioxide (SO_2_, in ug/m^3^) and nitrogen dioxide (NO_2_, in ug/m^3^).

Twin pregnancy and multiple pregnancies were excluded from this study. There were a total of 1,040,638 singleton live births from January 1st, 2005 to December 31st, 2011 in Shenzhen. The births with eligible gestational age (20–44 weeks) [30] accounted for 99.85 % of the whole data. The births whose mother’s LMP date was missing or implausible (<20 or >44 weeks) accounted for 0.15 % and were excluded from the analysis. Eligible live births with gestational age fewer than 37 weeks were considered preterm.

This study was approved by the medical ethics committee of Guangdong Women and Children Hospital. Data used in this study were anonymous and no individual identifiable information was available for the analysis.

### Statistical analysis

A distributed lag non-linear model (DLNM) was used to simultaneously investigate the non-linear and delayed effects of temperature on daily preterm. The count of daily PTBs typically follows a Poisson distribution [31–33]. Quasi-likelihood Poisson regression in a generalized linear model was used to model the natural logarithm of daily counts of PTB as functions of predictor variables. This methodology was based on a “cross-basis” function, which allowed the non-linear effect of daily preterm variation at each lag and the non-linear effects across lag days to be estimated [34, 35]. We initially constructed a “primary” model using the Akaike’s Information Creterion (AIC) to choose the df (knots) for daily average temperature and lag in the “primary” models, and we found that a cubic b-spline with 5 df for the daily mean temperature and 4 df in the lag space produced the best “primary” model with lowest AIC value [35]. Potential confounding factors were controlled for in the model, such as an indicator for day of week (DOW), an indicator for public holiday (PH), in order to control the seasonal and long-term trends and adjust for non-temperature aspects of weather, a natural cubic spline for day of the year (DOY, with df of 5/year), and a natural cubic spline of relative humidity (RH) and atmospheric pressure (BP) with the degrees of freedom (5 df), which was chosen by minimizing the AIC values [34, 36], and the linear function of air pollutants (NO_2_, PM_10_, SO_2_) were introduced into the model simultaneously. The daily number of pregnancies at risk for preterm birth with log(·) function (pregnancies between 20 and 36 weeks of gestation) was included in the model as offset [33, 37]. The model used for the analysis could be specified as follows:

$$ \begin{array}{l}\mathrm{Log}\left[\mathrm{E}\left(\mathrm{Y}\mathrm{t}\right)\right] = \upalpha + \mathrm{c}\mathrm{b}\ \left(\mathrm{Temp},\ 5,\ \mathrm{lag},\ 4\right) + \mathrm{ns}\ \left(\mathrm{R}\mathrm{H},\ 5\right) + \mathrm{ns}\ \left(\mathrm{B}\mathrm{P},\ 5\right) + \mathrm{N}{\mathrm{O}}_2 + \mathrm{P}{\mathrm{M}}_{10} + \\ {}\mathrm{S}{\mathrm{O}}_2 + \mathrm{ns}\ \left(\mathrm{D}\mathrm{O}\mathrm{Y},\ \mathrm{d}\mathrm{f} = 5/\mathrm{year}\right) + \upbeta 1*\mathrm{D}\mathrm{O}\mathrm{W} + \upbeta 2*\mathrm{P}\mathrm{H} + \upbeta 3* \log \left(\mathrm{Z}\mathrm{t}\right),\end{array} $$

where Yt denotes the observed daily preterm count on day t, α is the intercept, cb means the “cross-basis” function, ns(·) indicates a natural cubic splines for non-linear variables, Zt represents the daily number of pregnancies at risk for preterm birth on day t (pregnancies between 20 and 36 weeks of gestation), β is the regression coefficient.

In addition, we conducted sensitivity analysis: use of alternative degrees of freedom (4, 6 df/year) for temporal adjustment and change the degrees of freedom (4–7 df) for meteorological variables to evaluate the robustness of results.

According to a review [38], previous studies reported statistically significant association between the seasonal patterns and PTB. In order to test the validity of seasonal control in DLMN, we used a similar Poisson regression model as mentioned above and added month as a covariate into the model to compare the difference of the two models in controlling of season [39]. The model took the form:

$$ \mathrm{Log}\left[\mathrm{E}\left(\mathrm{Y}\mathrm{t}\right)\right] = \upalpha + \mathrm{c}\mathrm{b}\ \left(\mathrm{Temp},\ 5,\ \mathrm{lag},\ 4\right) + \mathrm{ns}\ \left(\mathrm{Month},\ 5\right) + \mathrm{ns}\ \left(\mathrm{R}\mathrm{H},\ 5\right) + \mathrm{ns}\ \left(\mathrm{B}\mathrm{P},\ 5\right) + \mathrm{N}{\mathrm{O}}_2 + \mathrm{P}{\mathrm{M}}_{10} + \mathrm{S}{\mathrm{O}}_2 + \mathrm{ns}\ \left(\mathrm{D}\mathrm{O}\mathrm{Y},\ \mathrm{d}\mathrm{f} = 5/\mathrm{year}\right) + \upbeta 1*\mathrm{D}\mathrm{O}\mathrm{W} + \upbeta 2*\mathrm{P}\mathrm{H} + \upbeta 3* \log \left(\mathrm{Z}\mathrm{t}\right). $$

Although an important advantage of the time-series design is the inherently controlling for the non-time-varying risk factors, even unknown or unrecorded [27]. In fact, the health effect of temperature is influenced by a variety of comprehensive factors including spatial distributions, exposure level, air pollution levels, and socio-demographic backgrounds. In the study of the association between the ambient temperature and PTB, the modification factors should be considered. The information on delivery mode, infant’s gender and maternal age are available in our database. By analyzing the potential modification effect of those variables on the association between temperature and PTB, it can help to identify the susceptible groups and estimate the degree of influence in different population and introduce more targeted public health interventions.

In order to completely capture the association of overall temperature on PTB and adjust for potential harvesting effect [40], we assumed a longer lag days, up to 30 days, between the exposure and preterm. We used the median of daily mean temperature of 24.5 °C as the reference temperature to report the relative risk (RR, with 95 % confidence intervals (CIs) of temperature (1st, 5th, 95th, and 99th percentiles of temperature) on PTB along specific lag days. This enabled us to obtain relative risk through the whole range of temperatures on different lag days. We also estimated the cumulative effects of low and high temperatures on preterm birth during lag periods (lag 0–30 days, where lag 0–30 represents the temperature on the day of preterm birth and the previous 30 days).

All statistical tests were two-sided and values of *P* < 0.05 were considered statistically significant. The DLNM package [35] in R software Version 3.0.2 (R Development Core Team, 2013) was utilized to fit all the models.

## Results

The distribution of daily singleton PTB, meteorological variables and air pollutants in Shenzhen were displayed in Table 1. The average number of daily singleton PTB in Shenzhen was 22.85. The average daily PTB was different according to different delivery modes, infant’s sex and maternal age. Among them, the age group of 20 ~ 34 years reported the highest number of the PTB cases (*n* = 18.94), the next is female daily PTB, 13.06, and then comes to vaginal delivery, 12.95. The mean temperature was 23.14 °C, and the temperature ranged from 5.40 to 32.70 °C during the study period. Shenzhen had an average relative humidity 70.89 %. Mean concentration of NO_2_, PM_10_ and SO_2_ were 46.37 g/m^3^, 61.11 g/m^3^, and 18.01 g/m^3^, respectively. And we also analyzed the lag effect of air pollutants on PTB (see Additional file 1: Table S1).

**Table 1:** Summary statistics of singleton PTBs, daily weather conditions and air pollutants in Shenzhen

Variable	Mean(SD)	Min	P25	P50	P75	Max
Temperature (°C)	23.14(5.56)	5.4	19.2	24.5	27.8	32.7
Humidity (%)	70.89(12.87)	17	65	73	80	99
Atmospheric pressure (hpa)	1006.07(6.61)	985.7	1001.1	1005.90	1010.70	1029.10
NO_2_ (μg/m^3^)	46.37(20.40)	10.67	31.77	41.75	56.38	166.13
PM_10_ (μg/m^3^)	61.11(32.19)	12.38	35.75	53.88	80.17	411.50
SO_2_ (μg/m^3^)	18.01(11.63)	2.88	9.50	15.06	22.96	105.67
All PTBs	22.85(7.98)	4	17	22	28	49
Maternal age 15 ~ 19 group PTBs	0.84(1.05)	0	0	1	1	6
Maternal age 20 ~ 34 group PTBs	18.94(7.10)	3	14	18	24	46
Maternal age 35 ~ 49 group PTBs	2.47(1.91)	0	1	2	4	12
Male PTBs	13.06(5.12)	1	9	12	16	33
Female PTBs	9.80(4.30)	1	7	9	12	26
Vaginal delivery PTBs	12.95(4.70)	1	10	13	16	29
Cesarean section PTBs	9.92(5.11)	1	6	9	13	30

Table 2 presented the PTB rate in Shenzhen from 2005 to 2011. There were a total of 58, 411 (5.61 % of total eligible births) singleton PTB over the study period. The PTB rate of cesarean section was 1.24 % higher than that of vaginal delivery. There was a higher percentage of PTB in boys compared with girls (5.90 % vs. 5.27 %), When stratified by maternal age, the maternal age 20 ~ 34 groups had the lowest PTB rate, which was 5.30 %.

**Table 2:** PTBs rate from 2005 to 2011 in Shenzhen

Items	Births	Proportion (%)	PTBs	Occurrence (%)
All	1,040,638	100	58,411	5.61
Year of births				
2005	94,335	9.07	5281	5.60
2006	115,428	11.09	6379	5.53
2007	146,934	14.12	7856	5.35
2008	163,138	15.68	8995	5.51
2009	158,953	15.27	8732	5.49
2010	174,515	16.77	10,071	5.77
2011	187,335	18.00	11,097	5.92
Maternal age				
Maternal age 15 ~ 19 group	28,491	2.74	2135	7.49
Maternal age 20 ~ 34 group	913,845	87.82	48,409	5.30
Maternal age 35 ~ 49 group	80,976	7.78	6326	7.81
Other (including missing or implausible)	17,326	1.66	1541	8.89
Sex of fetus				
Male	564,967	54.29	33,358	5.90
Female	475,535	45.70	25,038	5.27
Uncertain	136	0.01	15	11.03
Delivery mode				
Vaginal delivery	643,955	61.88	33,105	5.14
Cesarean section	396,683	38.12	25,306	6.38

In the sensitivity analyses, we adjusted the degrees of freedom (4, 6 df/year) for temporal adjustment and the degrees of freedom (4–7 df) for meteorological variables, and the effects estimates for temperature on PTB remained similar. HRs for temperature and PTB of the best model which chosen a natural cubic spline for day of the year (DOY, with df of 5/year), and a natural cubic spline of meteorological with the degrees of freedom (5 df) were presented on Tables 3 and 4. We added month as a covariate into the model, and the temperature effect estimates for PTB remained similar (see Additional file 1: Table S2 and S3).

**Table 3:** Maternal age categories relative risk (RR) and 95 % confidence intervals (CI) for total PTBs for temperature (1, 5, 95 and 99 % percentiles) at Different lag days with reference at 24.5 °C

	RR(95 % CI)			
	9 °C	12.5 °C	29.9 °C	30.7 °C
All PTBs				
Lag0	1.54(1.36–1.75)*	1.49(1.35–1.63)*	0.95(0.87–1.02)	0.96(0.88–1.05)
Lag5	1.03(1.00–1.07)*	1.05(1.03–1.07)*	0.99(0.98–1.01)	1.00(0.98–1.02)
Lag10	1.00(0.97–1.02)	1.01(0.99–1.02)	0.99(0.97–1.00)*	0.98(0.97–1.00)*
Lag15	0.99(0.97–1.01)	1.01(1.00–1.02)*	0.99(0.98–1.00)*	0.98(0.97–0.99)*
Lag20	1.00(0.98–1.01)	1.01(1.00–1.02)*	0.99(0.98–1.00)*	0.98(0.97–0.99)*
Lag25	1.01(1.00–1.03)*	1.01(1.00–1.02)*	0.99(0.98–1.00)*	0.98(0.97–0.99)*
Lag30	1.04(1.00–1.07)*	1.01(0.99–1.03)	0.99(0.97–1.00)*	0.98(0.96–1.00)*
Cumul	1.72(1.28–2.33)*	1.96(1.60–2.39)*	0.69(0.60–0.80)*	0.62(0.52–0.74)*
Maternal age 15 ~ 19 group PTBs				
Lag0	1.50(0.85–2.67)	1.62(1.06–2.49)*	1.03(0.74–1.42)	0.96(0.66–1.40)
Lag5	1.12(0.97–1.29)	1.07(0.98–1.17)	1.00(0.93–1.07)	1.01(0.93–1.10)
Lag10	1.00(0.90–1.12)	1.01(0.95–1.07)	0.97(0.93–1.02)	0.98(0.92–1.04)
Lag15	0.97(0.89–1.06)	1.00(0.96–1.05)	1.00(0.97–1.04)	1.01(0.96–1.05)
Lag20	0.98(0.90–1.07)	1.00(0.96–1.05)	1.02(0.98–1.05)	1.01(0.97–1.06)
Lag25	1.02(0.95–1.10)	1.01(0.97–1.06)	1.01(0.98–1.05)	1.00(0.96–1.04)
Lag30	1.07(0.92–1.25)	1.02(0.93–1.12)	1.01(0.94–1.08)	0.98(0.90–1.07)
Cumul	3.72(0.95–14.60)	2.74(1.15–6.52)*	1.04(0.56–1.94)	0.93(0.43–2.00)
Maternal age 20 ~ 34 group PTBs				
Lag0	1.61(1.40–1.85)*	1.54(1.39–1.71)*	0.95(0.87–1.03)	0.98(0.89–1.08)
Lag5	1.03(1.00–1.07)*	1.05(1.02–1.07)*	0.99(0.97–1.01)	0.99(0.97–1.01)
Lag10	1.00(0.98–1.03)	1.01(1.00–1.03)*	0.98(0.97–1.00)*	0.98(0.97–1.00)*
Lag15	0.99(0.97–1.01)	1.01(1.00–1.02)*	0.99(0.98–1.00)*	0.98(0.97–0.99)*
Lag20	0.99(0.97–1.01)	1.01(1.00–1.02)*	0.99(0.98–1.00)*	0.98(0.97–0.99)*
Lag25	1.01(0.99–1.03)	1.01(1.00–1.02)*	0.99(0.98–1.00)*	0.98(0.97–0.99)*
Lag30	1.03(1.00–1.07)*	1.01(0.98–1.03)	0.98(0.97–1.00)*	0.98(0.96–1.00)*
Cumul	1.74(1.25–2.42)*	2.02(1.63–2.51)*	0.70(0.60–0.82)*	0.64(0.53–0.78)*
Maternal age 35 ~ 49 group PTBs				
Lag0	1.53(1.10–2.13)*	1.49(1.16–1.91)*	0.92(0.75–1.13)	0.94(0.75–1.20)
Lag5	1.05(0.97–1.14)	1.09(1.04–1.15)*	1.02(0.98–1.07)	1.01(0.96–1.06)
Lag10	0.94(0.88–1.00)*	1.00(0.96–1.03)	1.00(0.96–1.03)	0.99(0.95–1.03)
Lag15	0.99(0.94–1.04)	1.01(0.98–1.04)	0.98(0.96–1.01)	0.98(0.95–1.01)
Lag20	1.02(0.97–1.07)	1.02(0.99–1.05)	0.98(0.96–1.00)*	0.98(0.95–1.01)
Lag25	1.03(0.99–1.08)	1.01(0.98–1.04)	0.99(0.97–1.01)	0.99(0.96–1.01)
Lag30	1.04(0.95–1.14)	1.00(0.95–1.05)	1.00(0.95–1.04)	1.00(0.95–1.05)
Cumul	1.86(0.86–4.04)	2.27(1.36–3.79)*	0.66(0.45–0.98)*	0.57(0.36–0.92)*

*P < 0.05

**Table 4:** Sex-specific and Delivery models relative risk (RR) and 95 % confidence intervals (CI) for total PTBs for temperature (1, 5, 95 and 99 % percentiles) at different lag days with reference at 24.5 °C

	RR(95 % CI)			
	9 °C	12.5 °C	29.9 °C	30.7 °C
Male PTBs				
Lag0	1.58(1.35–1.84)*	1.49(1.33–1.67)*	0.96(0.88–1.06)	0.98(0.88–1.09)
Lag5	1.05(1.01–1.09)*	1.06(1.03–1.08)*	1.00(0.98–1.02)	1.00(0.97–1.02)
Lag10	0.99(0.97–1.02)	1.01(0.99–1.02)	0.99(0.97–1.00)*	0.98(0.96–1.00)*
Lag15	0.99(0.96–1.01)	1.00(0.99–1.02)	0.99(0.98–1.00)*	0.98(0.97–1.00)*
Lag20	1.00(0.98–1.02)	1.00(0.99–1.02)	0.99(0.98–1.00)*	0.98(0.97–1.00)*
Lag25	1.02(1.00–1.04)*	1.01(1.00–1.02)*	0.99(0.98–1.00)*	0.98(0.97–0.99)*
Lag30	1.06(1.01–1.10)*	1.02(0.99–1.04)	0.99(0.97–1.02)	0.98(0.96–1.01)
Cumul	1.97(1.37–2.83)*	1.96(1.54–2.49)*	0.74(0.62–0.88)*	0.64(0.52–0.80)*
Female PTBs				
Lag0	1.49(1.25–1.77)*	1.48(1.30–1.68)*	0.93(0.84–1.03)	0.95(0.84–1.08)
Lag5	1.01(0.97–1.06)	1.04(1.01–1.06)*	0.99(0.97–1.01)	0.99(0.96–1.02)
Lag10	1.00(0.97–1.04)	1.01(0.99–1.03)	0.98(0.97–1.00)*	0.98(0.96–1.00)*
Lag15	0.99(0.96–1.02)	1.01(1.00–1.03)*	0.99(0.98–1.00)*	0.98(0.97–1.00)*
Lag20	0.99(0.96–1.02)	1.01(1.00–1.03)*	0.99(0.97–1.00)*	0.98(0.97–1.00)*
Lag25	1.00(0.98–1.02)	1.00(0.99–1.02)	0.98(0.97–0.99)*	0.98(0.97–0.99)*
Lag30	1.01(0.97–1.06)	0.99(0.96–1.02)	0.97(0.95–1.00)*	0.97(0.95–1.00)*
Cumul	1.40(0.92–2.12)	1.94(1.48–2.55)*	0.64(0.52–0.78)*	0.60(0.47–0.76)*
Vaginal delivery PTBs				
Lag0	1.36(1.18–1.57)*	1.29(1.16–1.44)*	0.93(0.85–1.01)	0.92(0.84–1.02)
Lag5	1.02(0.98–1.06)	1.03(1.01–1.05)*	0.99(0.98–1.01)	1.00(0.98–1.02)
Lag10	1.01(0.98–1.04)	1.02(1.00–1.03)*	0.98(0.97–1.00)*	0.98(0.96–0.99)*
Lag15	0.99(0.97–1.01)	1.01(1.00–1.02)*	0.99(0.98–1.00)*	0.98(0.97–1.00)*
Lag20	0.99(0.97–1.01)	1.00(0.99–1.02)	0.99(0.98–1.00)*	0.98(0.97–0.99)*
Lag25	1.00(0.98–1.02)	1.00(0.99–1.01)	0.99(0.98–1.00)*	0.98(0.96–0.99)*
Lag30	1.02(0.98–1.06)	0.99(0.97–1.02)	0.98(0.96–1.00)*	0.97(0.94–0.99)*
Cumul	1.58(1.12–2.22)*	1.67(1.33–2.10)*	0.69(0.58–0.81)*	0.58(0.48–0.71)*
Cesarean section PTBs				
Lag0	1.78(1.46–2.17)*	1.76(1.52–2.04)*	0.97(0.86–1.09)	1.02(0.89–1.18)
Lag5	1.06(1.00–1.11)*	1.07(1.04–1.10)*	0.99(0.97–1.02)	0.99(0.96–1.02)
Lag10	0.98(0.95–1.02)	1.00(0.98–1.02)	0.99(0.97–1.01)	0.99(0.97–1.01)
Lag15	0.98(0.96–1.01)	1.01(0.99–1.02)	0.98(0.97–1.00)*	0.98(0.96–1.00)*
Lag20	1.00(0.97–1.03)	1.01(1.00–1.03)*	0.98(0.97–1.00)*	0.98(0.96–1.00)*
Lag25	1.03(1.00–1.05)*	1.02(1.00–1.03)*	0.99(0.97–1.00)*	0.99(0.97–1.00)*
Lag30	1.06(1.00–1.12)*	1.02(0.99–1.05)	0.99(0.97–1.02)	1.00(0.96–1.03)
Cumul	1.93(1.21–3.08)*	2.33(1.71–3.16)*	0.70(0.56–0.88)*	0.68(0.51–0.90)*

*P < 0.05

**Fig. 2:**
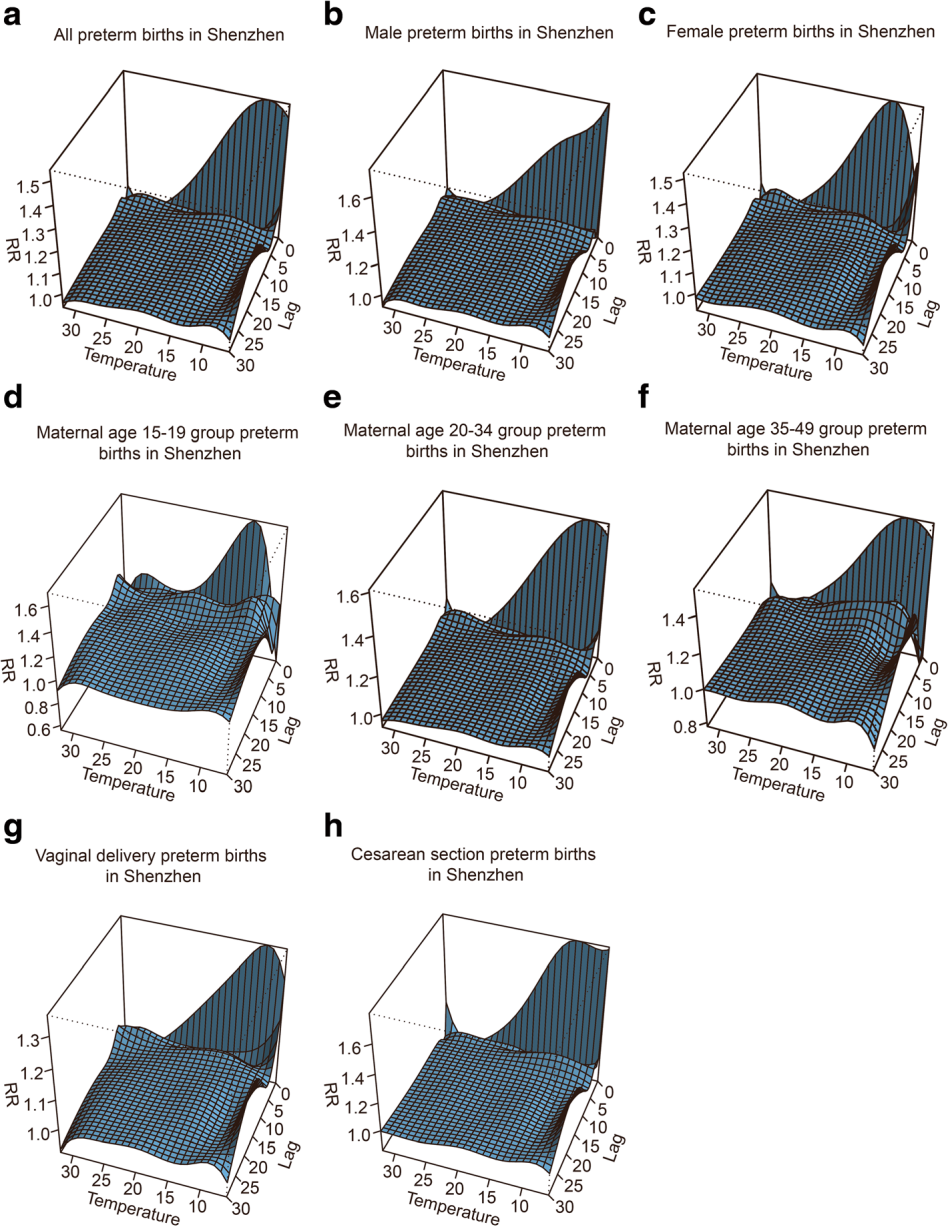
Three-D plot of RR along temperature and lags for PTB with reference at 24.5 °C by DLNM method (Panel **a** is for all preterm births; **b** is for male preterm births; **c** is female preterm births; **d** is for preterm births with maternal age of 15-19 years; **e** is for preterm births with maternal age of 20-34 years; **f** is for preterm births with maternal age of 35-49 years; **g** is for vaginal delivered preterm births; and **h** is for cesarean section preterm births)

The overall effect of temperature on PTB was illustrated in Fig. 2, showing a three-dimensional pattern of the RR along daily mean temperature and lag days (up to 30). The RR was calculated with the median of daily mean temperature 24.5 °C as the reference. The overall estimated association of temperature on PTB was non-linear. The figure represented different patterns of temperature effect on the risk of PTB depending on the modification indicator used. The RR estimates of the PTB for decreasing temperatures values followed a sharper pattern with several peaks of effect of variable magnitude according to temperature value and delay. When compared the result which added month as a covariate to further control of season (see Additional file 1: Figure S1) with Fig. 2, little difference was observed, and this result also indicated the validity of DLNM in controlling seasonal trend.

Table 3 described the relative risk of temperatures for preterm delivery at specific lag days (0, 5, 10, 15, 20, 25, and 30 days), and the temperatures (9.0, 12.5, 29.9, and 30.7 °C), which corresponded to the 1st, 5th, 95th, and 99th percentiles of the temperature distribution. It was found that for all of the singleton PTB, 9.0 °C and 12.5 °C were associated with an increased occurrence of PTBs, with 9 °C, RR = 1.54 (95 % CI: 1.36–1.75) exerted a stronger adverse effect than 12.5 °C, RR = 1.49 (95 % CI: 1.35–1.63) at lag 0. A positive association was also found with lag of 5, 25 to 30 days at 9 °C while the lag of 5, 15 to 25 days at 12.5 °C. The cumulative effect on PTB in 9 °C is lower than that in 12.5 °C, with RR = 1.72 (95 % CI: 1.28–2.33) and RR = 1.96 (95 %CI: 1.60–2.39), respectively. A negative association was found with lag of 10 to 30 days at 29.9 and 30.7 °C. The cumulative effects of high temperature (95th and 99th percentiles) on PTB are RR = 0.69 (95 % CI: 0.60–0.80) and RR = 0.62 (95 %CI: 0.52–0.74), respectively.

Table 3 also showed maternal age categories relative risk of the entire singleton preterm for temperatures (1st, 5th, 95th, and 99th percentiles) with reference at 24.5 °C at different days. The younger age (15 ~ 19 years) groups were only sensitive to low temperature (1st and 5th percentiles). In contrast, high temperature was negatively correlated with PTBs in the age (20 to 34 years) and (35 ~ 49 years) groups. The effects of 12.5 °C in the age groups of 15–19, 20–34, 35–49 years were RR = 1.62 (95 % CI: 1.06–2.49), RR = 1.54 (95 % CI: 1.39–1.71) and RR = 1.49 (95 % CI: 1.16–1.91) on the current day, respectively. The cumulative effects (30 days) of 12.5 °C in the age groups of 15 ~ 19, 20 ~ 34, 35 ~ 49 years were RR = 2.74 (95 % CI: 1.15–6.52), RR = 2.02 (95 % CI: 1.63–2.51) and RR = 2.27 (95 % CI: 1.36–3.79), respectively.

The protective effect of high temperature (95th and 99th percentiles) on the (20 ~ 34 years) groups was more endurable compared with the (35 ~ 49 years) groups. The cumulative effect at 30.7 °C was RR = 0.64 (95 % CI: 0.53–0.78) and RR = 0.57 (95 % CI: 0.36–0.92), respectively.

Table 4 presented relative risk of the singleton PTB for temperatures (1st, 5th, 95th, and 99th percentiles) by sex-specific and delivery mode categories at different days with reference at 24.5 °C. It showed that the association of the 1st percentile temperature (9.0 °C) in males was similar to that in females, RR = 1.58 (95 % CI: 1.35–1.84), RR = 1.49 (95 % CI: 1.25–1.77) on the current day, respectively.

A positive association on lag 0, lag 5, lag 25 and lag 30 at 9.0 °C was found in male preterm with the current day has the largest effect, RR = 1.58 (95 % CI: 1.35–1.84) while for female preterm, 9.0 °C was only correlated with PTBs increase on lag 0, with RR = 1.49 (95 % CI: 1.25–1.77). The positive association at 29.9 and 30.7 °C on female preterm was more endurable compared to the male preterm. For example, the RR for 29.9 °C was 0.97 (95 % CI: 0.95–1.00) on lag 30 in female preterm. Differences were found when stratifying the analysis by delivery mode, positive association of 1st and 5th percentiles of temperature on PTBs of cesarean section groups were more obvious than that of vaginal delivery groups. The effect on lag 0 with the RR = 1.78 (95 % CI: 1.46–2.17) in cesarean section preterm and RR = 1.36 (95 % CI: 1.18–1.57) in vaginal delivery preterm at 9.0 °C. The cumulative effects (up to 30 days) of 9 °C in cesarean section and vaginal delivery PTBs are RR = 1.93 (95 % CI: 1.21–308) and RR = 1.58 (95 % CI: 1.12–2.22), respectively A positive association on lag 5, 25 and lag 30 at 9.0 °C was also found in caesarian section preterm.

## Discussion

Along with the increasing temperatures associated with climate change, interests have been increasing to examine the association between high temperature and human health, including preterm birth, such as researches reported by Liajinian [16], Porter [26], Yackerson [18], Basu [17], Dadvand [20], Strand [22], and Vicedo-Cabrera [21]. The previous studies [31] supported the association between the high temperature and the PTB. However, our study identified that in Shenzhen, high temperatures (95th and 99th percentiles) appears to be a protective factor on the PTB.

The protective effect of high temperature observed in this study was contrasting to a few previous studies. The potential biological mechanism remained largely unknown. Shenzhen is a subtropical and coastal city, even during the summer season, the high temperature is not as high as that in other cities. The average temperature was 23.14 °C, and the 95th percentile of daily mean temperature was 29.9 °C in Shenzhen during the study period. For example, in the studies in California, the average temperature was 31.5 °C and the 95th percentile temperature was 37.1 °C [17]. Dadvand’s study in Spain had a temperature range of 27.9 ~ 38.8 °C, and the 95th percentile of temperature was 30.4 °C [20]. Shenzhen is a developed city with highest social-economic status, most people living in this city have access to air conditioner, so the exposure to higher temperature in summer was minimal, especially for the pregnant women.

A few studies have examined the association between the low temperatures and the PTB in developed countries. For example, studies in London and Rome did not find significant association between low temperatures and PTB [24, 27], while the Sweden study found that cold temperature was associated with an increased risk of PTB [25], which is consistent with our study. In our study, the low temperatures (1st and 5th percentiles) were significantly associated with increased risk of the PTB, and the strongest association was observed for lag 0 at 9 °C (5th percentile), which means much greater magnitude of cold temperature effect at lag 0, compared to cold temperature effects at greater lags. Shenzhen is located in low latitudes. According to prior studies, although the populations in low latitude have fewer opportunities to exposure to low temperatures, they have a low adaptation to low ambient temperature [41]. Compared with populations in high latitudes, they are more sensitive to the low temperatures [42, 43]. The humid and cold weather in Shenzhen has more harmful effects on physiology compared with the dry and cold weather in other high latitude areas (London is in 51.5° north latitude, and Rome is in 42° north latitude). Shenzhen is in southeast of China, the residents do not routinely use heat radiators in winter, so they are exposed to a colder temperatures indoors in winter [44].

Only two previous studies [17, 22] considered whether the temperature was related to the sex of a preterm infant, and these studies did not report a significant association. Our findings are consistent with the two studies. Our study showed the association between the low temperature and the male PTB is similar to that of the female PTB while the protective effect of the high temperature in avoiding female PTB is more endurable.

We found a significant association of the low temperature with the risk of PTB in younger age (15 ~ 19 years) groups, indicating they were more susceptible to PTB. For the younger mothers, they may be in poor nutrition, poor physical status or have limited self-protection awareness and insufficient health service which may trigger PTB [45, 46]. These early findings suggested that the health information about reducing exposure to cold temperatures should be emphasized in younger women. With this regard, more researches in other populations using different study design should be undertaken to determine the strength of the association and provide stronger evidence for this focused effort.

Recently, there was a report about the ambient temperature and the risk of preterm birth in Guangzhou, China. It showed that the cold temperature was associated with an increased risk of PTB [47]. Our finding was in accordance with this study. However, the high temperature might be a protective factor of the PTB in Shenzhen while it is a risk factor in Guangzhou. This result might be explained by the fact that the high temperature in Shenzhen is lower than that of Guangzhou, the 99th percentile is 30.7 and 31.9 °C respectively. And urban heat island effect in Guangzhou is more severe than that of Shenzhen [48]. Our study further classified singleton PTBs cohort according to different genders and maternal ages and analyzed the association of temperature on PTBs in those subgroups, which helps identify the susceptible groups and estimate the degree of influence in different subgroups. For the newborns in our study, the cesarean section presented 38.12 %, and cesarean section PTBs presented 43.32 % of all the PTBs. Our study also divided PTBs into the vaginal delivery and cesarean section cohort, which can be more comprehensive to grasp the influence of the temperature for all the PTBs. However, there is a limitation that the data were not available on whether preterm labour was spontaneous or induced.

This is a large-scale and population-based epidemiologic study of the association between the temperature and the PTB in China. Much of the prior evidence for the PTB from the environment exposures were based on air pollution [32, 33, 49, 50], and studies reported statistically significant association between the seasonal patterns and preterm birth [38, 51–54]. In this study, the variables of the air pollution and the seasonality of birth were controlled and hopefully negated any potential confounding by them. On the other hand, a few limitations should be considered when interpreting findings from our study. First, in data collection, we used environmental monitoring data to represent the individual exposure level to weather conditions, which might not accurately reflect the real individual exposure. This exposure misclassification may be more important for the high temperatures than the low temperatures, due to the widespread use of air conditioners, but the limited use of heating devices may explain the significant associations between the high temperatures and the PTB identified by other studies. Second, we had to rely on the data provided on the birth certificates, and we did not have the information on several characteristics such as the socio-demographic and maternal health condition, thus we could not account for these variables in our analyses.

## Conclusions

In Shenzhen, the low temperatures appeared to be the risk factor for the PTB, and the high temperatures might be a protective factor of the PTB. Differences in results of the delivery mode reflect differences in population susceptibility.

## Abbreviations

AIC, Akaike’s information criterion; BP, barometric pressure; CI, confidence interval; df, the degrees of freedom; DLNM, distributed lag non-linear model; DOW, day of the week; DOY, day of the year; PH, public holiday; PM_10_, particulate matter with aerodynamic diameter ≤10 μm; PTB, preterm birth; RH, relative humidity; RR/RR(s), relative risk(s)

## Additional file

Additional file 1: Table S1. Results of the lag effect of air pollutants (NO_2_, PM_10_ and SO_2_) on preterm birth. **Table S2.** Maternal age categories relative risk (RR) and 95 % confidence intervals (CI) for total PTBs with month control for temperature (1, 5, 95 and 99 % percentiles) at Different lag days with reference at 24.5 °C. **Table S3.** Sex-specific and Delivery models relative risk (RR) and 95 % confidence intervals (CI) for total PTBs with month control for temperature (1, 5, 95 and 99 % percentiles) at different lag days with reference at 24.5 °C. **Figure S1.** Three-D plot of RR along temperature and lags for PTB with reference at 24.5 °C by further control of month. (DOC 15271 kb)
