# Supplementary material for: The association between ambient temperature and preterm birth in Shenzhen, China: a distributed lag non-linear time series analysis
Source: Environ Health. 2016 Aug 8;15:84. doi: 10.1186/s12940-016-0166-4 (PMC4977688; doi:10.1186/s12940-016-0166-4)

**The Supplementary material: Supplementary tables of association between air pollution and preterm**

## Table S1 Results of the lag effect of air pollutants (NO_2_, PM_10_ and SO_2_) on preterm birth

| Lag days | NO_2_ | PM_10_ | SO_2_ |
| --- | --- | --- | --- |
|  | RR(95% CI) | RR(95% CI) | RR(95% CI) |
| Lag0 | 1.005(0.999-1.011) | 0.998(0.994-1.002) | 1.003(0.991-1.015) |
| Lag1 | 1.003(0.998-1.008) | 1.002(0.999-1.006) | 1.008(0.997-1.018) |
| Lag2 | 1.000(0.996-1.003) | 1.001(0.999-1.003) | 1.001(0.995-1.008) |
| Lag3 | 0.999(0.995-1.003) | 1.000(0.998-1.003) | 0.998(0.990-1.006) |
| Lag4 | 1.002(0.999-1.006) | **1.002(1.000-1.004)*** | 1.002(0.996-1.009) |
| Lag5 | 1.004(0.999-1.009) | 1.003(0.999-1.006) | 1.008(0.998-1.018) |
| Lag6 | 0.995(0.990-1.001) | 0.995(0.990-0.999) | 0.998(0.986-1.009) |

Notes：*P＜0.05

When a 10 ug/m^3^ increase，PM_10_ concentrations have lag effect on preterm birth on lag 4, with effect RR of 1.002 (95% CI: 1.000-1.004).

## Table S2 Maternal age categories relative risk (RR) and 95% confidence intervals (CI) for total PTBs with month control for temperature (1%, 5%, 95% and 99% percentiles) at Different lag days with reference at 24.5℃

|  | RR(95% CI) | | | |
| --- | --- | --- | --- | --- |
|  | 9℃ | 12.5℃ | 29.9℃ | 30.7℃ |
| **All PTBs** | | | | |
| Lag0 | **1.55(1.36-1.76)*** | **1.49(1.35-1.64)*** | 0.94(0.87-1.02) | 0.96(0.88-1.05) |
| Lag5 | **1.03(1.00-1.07)*** | **1.05(1.03-1.07)*** | 0.99(0.98-1.01) | 1.00(0.98-1.02) |
| Lag10 | 1.00(0.97-1.02) | 1.01(0.99-1.02) | **0.98(0.97-1.00)*** | **0.98(0.97-1.00)*** |
| Lag15 | 0.99(0.97-1.01) | **1.01(1.00-1.02)*** | **0.99(0.98-1.00)*** | **0.98(0.97-0.99)*** |
| Lag20 | 1.00(0.98-1.01) | **1.01(1.00-1.02)*** | **0.99(0.98-1.00)*** | **0.98(0.97-0.99)*** |
| Lag25 | **1.01(1.00-1.03)*** | **1.01(1.00-1.02)*** | **0.99(0.98-1.00)*** | **0.98(0.97-0.99)*** |
| Lag30 | **1.04(1.00-1.07)*** | 1.01(0.99-1.03) | **0.99(0.97-1.00)*** | **0.98(0.96-1.00)*** |
| Cumul | **1.73(1.28-2.34)*** | **1.97(1.61-2.40)*** | **0.69(0.60-0.80)*** | **0.62(0.52-0.74)*** |
| **Maternal age 15**~**19 group PTBs** | | | | |
| Lag0 | 1.56(0.87-2.76) | **1.66(1.08-2.55)*** | 1.03(0.74-1.42) | 0.96(0.66-1.40) |
| Lag5 | 1.12(0.97-1.28) | 1.07(0.98-1.17) | 1.00(0.93-1.07) | 1.01(0.93-1.10) |
| Lag10 | 1.00(0.90-1.11) | 1.01(0.95-1.07) | 0.97(0.93-1.03) | 0.97(0.92-1.04) |
| Lag15 | 0.98(0.90-1.07) | 1.00(0.96-1.05) | 1.00(0.97-1.04) | 1.01(0.96-1.05) |
| Lag20 | 0.99(0.91-1.07) | 1.01(0.96-1.05) | 1.02(0.98-1.05) | 1.01(0.97-1.06) |
| Lag25 | 1.02(0.95-1.10) | 1.01(0.97-1.06) | 1.01(0.98-1.05) | 1.00(0.96-1.04) |
| Lag30 | 1.06(0.91-1.24) | 1.02(0.93-1.11) | 1.01(0.94-1.08) | 0.98(0.90-1.07) |
| Cumul | 3.74(0.95-14.77) | **2.77(1.16-6.60)*** | 1.04(0.56-1.94) | 0.93(0.43-2.00) |
| **Maternal age 20**~**34 group PTBs** | | | | |
| Lag0 | **1.61(1.40-1.85)*** | **1.54(1.39-1.71)*** | 0.95(0.87-1.03) | 0.97(0.88-1.07) |
| Lag5 | **1.03(1.00-1.07)*** | **1.05(1.03-1.07)*** | 0.99(0.97-1.01) | 0.99(0.97-1.02) |
| Lag10 | 1.00(0.98-1.03) | **1.01(1.00-1.03)*** | **0.98(0.97-1.00)*** | **0.98(0.96-1.00)*** |
| Lag15 | 0.99(0.97-1.01) | **1.01(1.00-1.02)*** | **0.99(0.98-1.00)*** | **0.98(0.97-0.99)*** |
| Lag20 | 0.99(0.97-1.01) | **1.01(1.00-1.02)*** | **0.99(0.98-1.00)*** | **0.98(0.97-0.99)*** |
| Lag25 | 1.01(0.99-1.03) | **1.01(1.00-1.02)*** | **0.99(0.98-1.00)*** | **0.98(0.97-0.99)*** |
| Lag30 | **1.03(1.00-1.07)*** | 1.01(0.98-1.03) | **0.98(0.97-1.00)*** | **0.98(0.96-1.00)*** |
| Cumul | **1.75(1.26-2.43)*** | **2.03(1.64-2.53)*** | **0.70(0.60-0.82)*** | **0.64(0.53-0.78)*** |
| **Maternal age 35~49 group PTBs** | | | | |
| Lag0 | **1.54(1.10-2.14)*** | **1.49(1.16-1.92)*** | 0.92(0.75-1.13) | 0.94(0.74-1.19) |
| Lag5 | 1.05(0.97-1.14) | **1.09(1.04-1.15)*** | 1.02(0.98-1.07) | 1.01(0.96-1.07) |
| Lag10 | **0.94(0.88-1.00)*** | 1.00(0.96-1.03) | 0.99(0.96-1.03) | 0.99(0.95-1.02) |
| Lag15 | 0.99(0.94-1.04) | 1.01(0.98-1.04) | 0.98(0.96-1.01) | 0.98(0.95-1.01) |
| Lag20 | 1.02(0.97-1.07) | 1.02(0.99-1.05) | 0.98(0.96-1.01) | 0.98(0.95-1.01) |
| Lag25 | 1.03(0.99-1.08) | 1.01(0.99-1.04) | 0.99(0.97-1.01) | 0.99(0.96-1.01) |
| Lag30 | 1.04(0.94-1.13) | 1.00(0.95-1.05) | 1.00(0.95-1.04) | 1.00(0.94-1.05) |
| Cumul | 1.87(0.86-4.07) | **2.27(1.36-3.78)*** | **0.66(0.45-0.97)*** | **0.57(0.35-0.92)*** |

**P*<0.05

## Table S3 Sex-specific and Delivery models relative risk (RR) and 95% confidence intervals (CI) for total PTBs with month control for temperature (1%, 5%, 95% and 99% percentiles) at different lag days with reference at 24.5℃

|  | RR(95% CI) | | | |
| --- | --- | --- | --- | --- |
|  | 9℃ | 12.5℃ | 29.9℃ | 30.7℃ |
| **Male PTBs** | | | | |
| Lag0 | **1.58(1.36-1.85)*** | **1.49(1.33-1.68)*** | 0.96(0.87-1.05) | 0.97(0.87-1.08) |
| Lag5 | **1.05(1.01-1.09)*** | **1.06(1.03-1.08)*** | 1.00(0.98-1.02) | 1.00(0.98-1.02) |
| Lag10 | 0.99(0.97-1.02) | 1.01(0.99-1.02) | **0.98(0.97-1.00)*** | **0.98(0.96-1.00)*** |
| Lag15 | 0.99(0.96-1.01) | 1.00(0.99-1.02) | **0.99(0.98-1.00)*** | **0.98(0.97-1.00)*** |
| Lag20 | 1.00(0.98-1.02) | 1.00(0.99-1.02) | **0.99(0.98-1.00)*** | **0.98(0.97-1.00)*** |
| Lag25 | **1.02(1.00-1.04)*** | **1.01(1.00-1.02)*** | **0.99(0.98-1.00)*** | **0.98(0.97-0.99)*** |
| Lag30 | **1.06(1.01-1.10)*** | 1.02(0.99-1.04) | 0.99(0.97-1.01) | **0.98(0.96-1.00)*** |
| Cumul | **1.97(1.37-2.83)*** | **1.96(1.54-2.49)*** | **0.74(0.62-0.88)*** | **0.64(0.52-0.79)*** |
| **Female PTBs** | | | | |
| Lag0 | **1.49(1.25-1.77)*** | **1.47(1.29-1.67)*** | 0.93(0.83-1.03) | 0.95(0.84-1.07) |
| Lag5 | 1.01(0.97-1.06) | **1.04(1.01-1.07)*** | 0.99(0.97-1.01) | 0.99(0.97-1.02) |
| Lag10 | 1.00(0.97-1.04) | 1.01(0.99-1.03) | **0.98(0.97-1.00)*** | **0.98(0.96-1.00)*** |
| Lag15 | 0.99(0.96-1.02) | **1.01(1.00-1.03)*** | **0.99(0.98-1.00)*** | **0.98(0.97-1.00)*** |
| Lag20 | 0.99(0.97-1.02) | **1.01(1.00-1.03)*** | **0.99(0.97-1.00)*** | **0.98(0.97-1.00)*** |
| Lag25 | 1.00(0.98-1.02) | 1.00(0.99-1.02) | **0.98(0.97-0.99)*** | **0.98(0.97-0.99)*** |
| Lag30 | 1.01(0.97-1.06) | 0.99(0.96-1.02) | **0.97(0.95-1.00)*** | **0.97(0.95-1.00)*** |
| Cumul | 1.41(0.93-2.13) | **1.96(1.49-2.58)*** | **0.64(0.53-0.78)*** | **0.60(0.47-0.76)*** |
| **Vaginal delivery PTBs** | | | | |
| Lag0 | **1.36(1.18-1.57)*** | **1.29(1.16-1.44)*** | 0.92(0.85-1.01) | 0.92(0.82-1.02) |
| Lag5 | 1.02(0.98-1.05) | **1.03(1.01-1.05)*** | 1.00(0.98-1.01) | 1.00(0.98-1.02) |
| Lag10 | 1.01(0.98-1.04) | **1.02(1.00-1.03)*** | **0.98(0.97-0.99)*** | **0.98(0.96-0.99)*** |
| Lag15 | 0.99(0.97-1.01) | **1.01(1.00-1.02)*** | **0.99(0.98-1.00)*** | **0.98(0.97-1.00)*** |
| Lag20 | 0.99(0.97-1.01) | 1.00(0.99-1.02) | **0.99(0.98-1.00)*** | **0.98(0.97-1.00)*** |
| Lag25 | 1.00(0.98-1.02) | 1.00(0.99-1.01) | **0.99(0.98-1.00)*** | **0.98(0.96-0.99)*** |
| Lag30 | 1.02(0.98-1.06) | 1.00(0.97-1.02) | **0.98(0.96-1.00)*** | **0.96(0.94-0.99)*** |
| Cumul | **1.57(1.12-2.22)*** | **1.68(1.34-2.11)*** | **0.68(0.58-0.80)*** | **0.58(0.47-0.70)*** |
| **Cesarean section PTBs** | | | | |
| Lag0 | **1.79(1.47-2.18)*** | **1.77(1.53-2.05)*** | 0.96(0.85-1.09) | 1.02(0.88-1.17) |
| Lag5 | **1.06(1.01-1.11)*** | **1.07(1.04-1.10)*** | 0.99(0.97-1.02) | 0.99(0.96-1.03) |
| Lag10 | 0.98(0.95-1.02) | 1.00(0.98-1.02) | 0.99(0.97-1.01) | 0.99(0.96-1.01) |
| Lag15 | 0.98(0.96-1.01) | 1.01(0.99-1.02) | **0.98(0.97-1.00)*** | **0.98(0.96-1.00)*** |
| Lag20 | 1.00(0.97-1.03) | **1.01(1.00-1.03)*** | **0.98(0.97-1.00)*** | **0.98(0.96-1.00)*** |
| Lag25 | **1.03(1.00-1.05)*** | **1.02(1.00-1.03)*** | **0.99(0.97-1.00)*** | **0.99(0.97-1.00)*** |
| Lag30 | **1.06(1.00-1.12)*** | 1.02(0.99-1.05) | 0.99(0.97-1.02) | 1.00(0.96-1.03) |
| Cumul | **1.95(1.22-3.12)*** | **2.33(1.72-3.17)*** | **0.70(0.56-0.88)*** | **0.68(0.51-0.90)*** |

**P*<0.05

## The Supplementary material: Supplementary figure of association between air pollution and preterm

Figure S1 Three-D plot of RR along temperature and lags for PTB with reference at 24.5℃ by further control of month

**Figure S1 Legend**

Figure S1 showed the result which added month as a covariate to further control of season. Comparing Figure 2 with Figure S1, when the month was controlled, little difference was observed, and this result also indicated the validity of DLNM in controlling seasonal trend.

**Figure S1 Three-D plot of RR along temperature and lags for PTB with reference at 24.5℃ by further control of month**


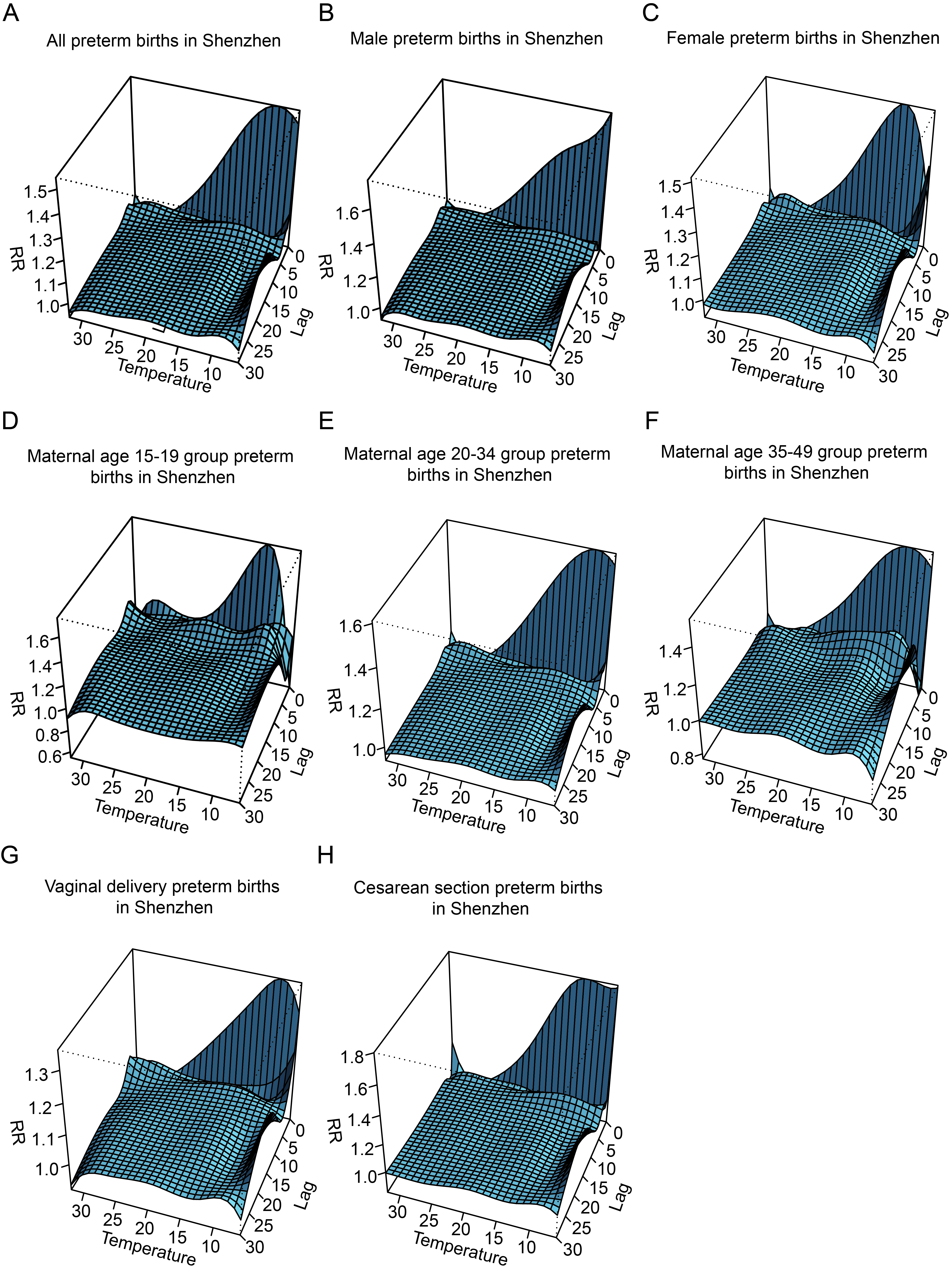

Supplement: Additional file 1: Table S1. — Results of the lag effect of air pollutants (NO2, PM10 and SO2) on preterm birth. Table S2. Maternal age categories relative risk (RR) and 95 % confidence intervals (CI) for total PTBs with month control for temperature (1, 5, 95 and 99 % percentiles) at Different lag days with reference at 24.5 °C. Table S3. Sex-specific and Delivery models relative risk (RR) and 95 % confidence intervals (CI) for total PTBs with month control for temperature (1, 5, 95 and 99 % percentiles) at different lag days with reference at 24.5 °C. Figure S1. Three-D plot of RR along temperature and lags for PTB with reference at 24.5 °C by further control of month. (DOC 15271 kb) [file 12940_2016_166_MOESM1_ESM.doc]
